# Deep learning-based approach for phenotypic trait extraction and computation of tomato under varying water stress

**DOI:** 10.3389/fpls.2025.1660593

**Published:** 2025-09-15

**Authors:** Weiyue Yang, Jinshan Li, Yayang Feng, Xuemin Li, Rui Zheng, Xiulu Sun

**Affiliations:** ^1^ Institute of Farmland Irrigation, Chinese Academy of Agricultural Sciences, Xinxiang, China; ^2^ Graduate School of Chinese Academy of Agricultural Sciences, Beijing, China; ^3^ Key Laboratory of Water-Saving Agriculture of Henan Province, Xinxiang, China

**Keywords:** water stress, visible light images, deep learning, phenotype calculation, tomato

## Abstract

**Introduction:**

With the advancement of imaging technologies, the efficiency of acquiring plant phenotypic information has significantly improved. The integration of deep learning has further enhanced the automatic recognition of plant structures and the accuracy of phenotypic parameter extraction. To enable efficient monitoring of tomato water stress, this study developed a deep learning-based framework for phenotypic trait extraction and parameter computation, applied to tomato images collected under varying water stress conditions.

**Methods:**

Based on the You Only Look Once version 11 nano (YOLOv11n) object detection model, adaptive kernel convolution (AKConv) was integrated into the backbone’s C3 module with kernel size 2 convolution (C3k2), and a recalibration feature pyramid detection head based on the P2 layer was designed.

**Results and discussion:**

Results showed that the improved model achieved a 4.1% increase in recall, a 2.7% increase in mAP50, and a 5.4% increase in mAP50–95 for tomato phenotype recognition. Using the bounding box information extracted by the model, key phenotype parameters were further calculated through geometric analysis. The average relative error for plant height was 6.9%, and the error in petiole count was 10.12%, indicating good applicability and accuracy for non-destructive crop phenotype analysis. Based on these extracted traits, multiple sets of weighted combinations were constructed as input features for classification. Seven classification algorithms—Logistic Regression, Support Vector Machine, Random Forest, Decision Tree, K-Nearest Neighbors, Naive Bayes, and Gradient Boosting—were used to differentiate tomato plants under different water stress conditions. The results showed that Random Forest consistently performed the best across all combinations, with the highest classification accuracy reaching 98%. This integrated approach provides a novel approach and technical support for the early identification of water stress and the advancement of precision irrigation.

## Graphical Abstract

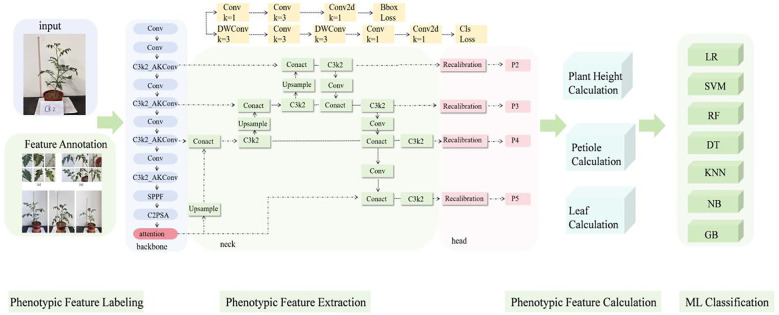

## 1 Introduction

Smart irrigation systems have increasingly become a key strategy for improving water use efficiency and enhancing crop productivity. Currently, such systems primarily rely on the integration of soil moisture, meteorological data, and intelligent algorithms to dynamically respond to crop water demands (Mason et al., 2019; Jimenez et al., 2020). However, such irrigation strategies are limited under complex field conditions. For instance, soil-moisture-based irrigation control is highly susceptible to interference from factors such as salinity and root distribution, thereby reflecting only localized water status rather than the plant’s actual physiological water needs. Similarly, irrigation methods based on evapotranspiration or historical weather data often fail to accommodate the dynamic water requirements of crops at different growth stages, potentially leading to over irrigation or under irrigation (Jamroen et al., 2020; Touil et al., 2022). Furthermore, these approaches tend to overlook phenotypic changes in crops, making it difficult to accurately detect early signs of stress and thereby compromising the timeliness and precision of irrigation management.

Recently, with the significant progress of applying image recognition technologies in agriculture, the potential for early identification of crop water stress and monitoring of physiological status proposes a new methodology and a novel perspective. Image recognition, as a non-contact, high-efficiency, and highly automated monitoring method (Cen et al., 2020; Zhang et al., 2020), has gradually become an important tool for acquiring phenotypic data in agricultural research (Wang and Su, 2022; Kim, 2024). Studies have shown that functional phenotyping methods from complex system architectures, strong dephenotyping methods can capture real-time physiological responses of plants under drought conditions, enabling more accurate assessment of drought tolerance (Hein et al., 2021; Mansoor and Chung, 2024). Meanwhile, continuous advancements in field-based high-throughput phenotyping platforms and multimodal imaging technologies have enabled large-scale, multidimensional analyses of crop stress responses under realistic agricultural environments (Langan et al., 2022). These developments provide a solid technical foundation for promoting the application of image-based technologies in smart irrigation.

However, effectively applying image recognition in irrigation management and crop water stress detection remains challenging, especially under complex field conditions and suboptimal imaging environments. On one hand, Phenotyping methods often rely on 3D reconstruction or manual measurements (Tong, 2022; Li, 2023). which, although capable of extracting morphological traits to some extent, suffependence on specialized hardware, cumbersome processing workflows, and high deployment costs. On the other hand, plants often exhibit complex structures and small-scale targets under water stress conditions, which are easily affected by variable lighting and background noise—reducing the precision and stability of feature extraction. While technologies such as multi-view stereo imaging and structured light laser scanning perform well in generating point clouds and measuring panicle height, their high sensitivity to lighting and plant motion, as well as their costly equipment, limit their practical use in field environments (Mohamed and Dudley, 2019). Moreover, most current plant phenotyping studies remain confined to controlled environments, and field-based phenotyping still faces significant challenges in image acquisition quality (Perez-Sanz et al., 2017) and real-time data processing (Cao et al., 2021).

To address the aforementioned challenges, researchers have increasingly shifted toward phenotyping approaches that integrate image recognition and deep learning. For instance, Aich (Aich and Stavness, 2017) proposed a deep neural network-based method for leaf counting, enabling automatic identification and quantification of plant leaves. Attri (Attri et al., 2023) integrated thermal imaging, image processing, and deep learning to successfully detect drought stress in maize, enabling efficient monitoring of crop water status. Dong (Dong, 2024) utilized Micro-CT imaging combined with deep learning to extract high-precision features of regenerated rice stems. Zou (Zou, 2024) estimated tomato plant height and canopy structure through image processing and 3D reconstruction, effectively addressing the limitations of manual methods in capturing spatial plant architecture. These studies highlight the broad applicability of image recognition in quantifying structural phenotypes in plants. On this basis, the integration of image recognition and deep learning has further accelerated the intelligent transformation of phenotypic analysis, making it feasible to automatically identify structural traits of plants (Kharraz and Szabó, 2023). For example, Li (Li et al., 2025) developed the MARS-Phenobot system to achieve high-throughput measurement of fruit-related traits such as blueberry yield, maturity, and firmness, effectively reducing the reliance on manual phenotyping. Similarly, Wang (Wang, 2021) constructed a deep learning model based on VGG16-SSD to automatically measure tomato plant height, addressing the low efficiency and high error rates associated with traditional methods. These studies provide reliable data for uncovering physiological responses and structural changes during plant growth, promoting the advancement of plant phenotyping analysis under stress conditions.

Among various deep learning models, the You Only Look Once (YOLO) model has gained considerable attention in agricultural contexts due to its excellent real-time performance and detection accuracy. The model effectively identifies small objects and complex plant structures while maintaining high inference speed, making it particularly well-suited for scenarios with dense, overlapping plant components. YOLO predicts both the spatial location and class of targets in the form of bounding boxes—a fundamental approach widely used in object detection tasks. It enables automated, bounding-box-level detection and parameter extraction for structural phenotypic traits such as leaves, petioles, and plant height. For example, He (He, 2023) developed a soybean pod detection and weight estimation system by integrating an improved YOLOv5 model and BP neural network, enhancing the efficiency of trait data acquisition. Xiang (Xiang, 2022) applied YOLOX to achieve automatic detection and counting of strawberries, improving the automation and accuracy of fruit-level phenotyping. These studies demonstrate that integrating deep learning models with phenotypic trait extraction can not only reduce manual intervention but also significantly enhance measurement efficiency and data consistency (Dong et al., 2022).Therefore, YOLO-based structural trait detection presents a promising path for efficiently identifying plant responses to water stress.

Under drought-induced water stress conditions, the rapid and accurate extraction of plant phenotypic traits is crucial for the implementation of smart irrigation strategies. However, research on the structural phenotypic changes of tomato plants under varying water stress conditions remains limited. Tomato plants present unique challenges for phenotypic analysis due to their small target size, compact structure, and significant morphological responses under drought conditions. Therefore, there is still a lack of automated methods that are both highly accurate and adaptable to field environments for identifying the dynamic phenotypic responses of tomato plants under drought stress. To address this gap, the study aims to develop a high-performance object detection model based on YOLOv11 for the automatic recognition and quantification of key tomato phenotypic traits under multiple water stress conditions. This approach is expected to provide technical support for drought response analysis and smart irrigation management. The specific objectives of this study are as follows: 1) To develop an improved YOLOv11n-based model for the automatic identification and precise quantification of key structural traits in tomato plants, including plant height, number of petioles, and number of leaves, with enhanced detection capability for small objects and multi-scale features; 2) To establish an efficient phenotypic parameter extraction framework capable of synchronously extracting multiple traits, in order to assess the impact of water stress on tomato phenotypes and ensure both accuracy and practical applicability in parameter measurement.

## 2 Materials and methods

### 2.1 The potted experiment

**Figure 1:**
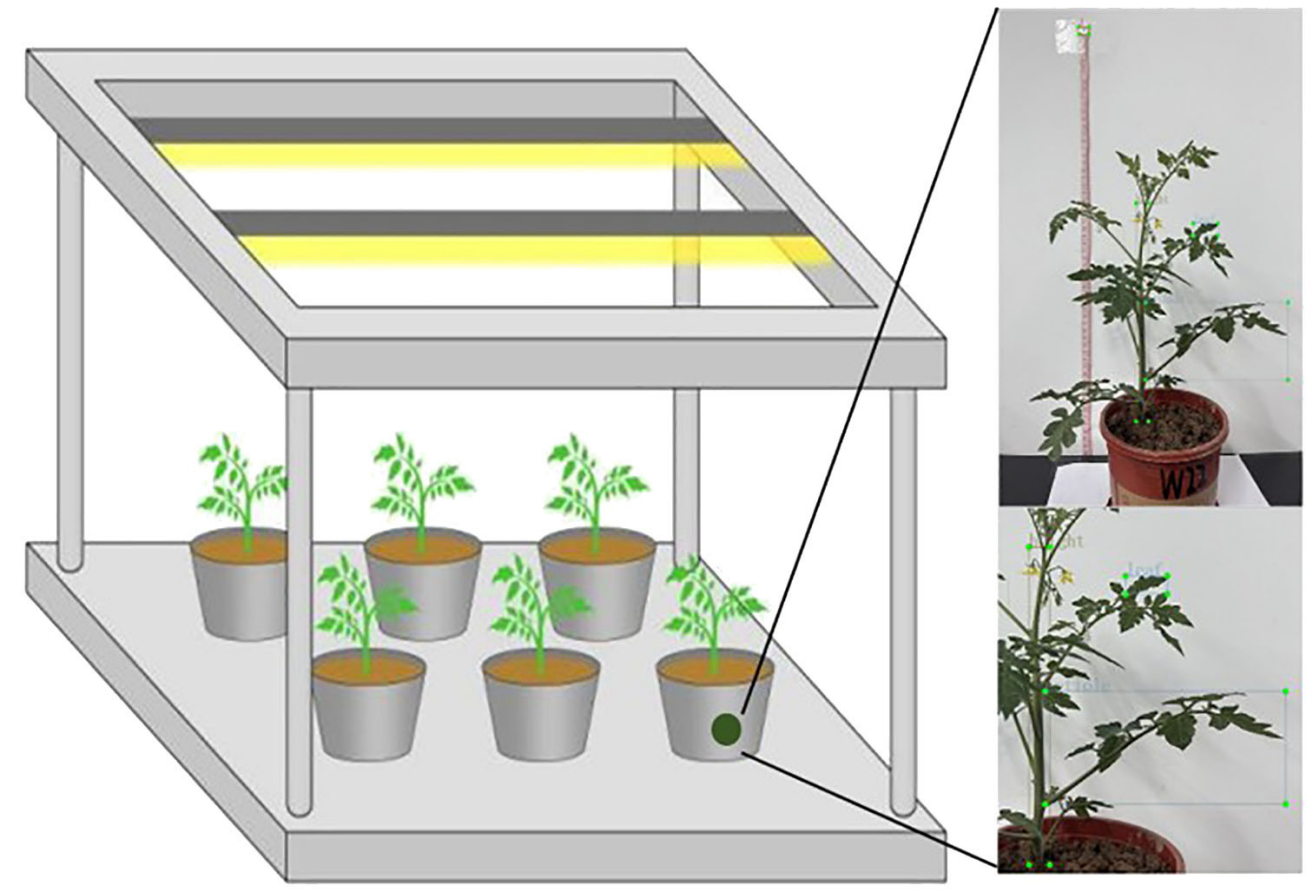
Layout of the experimental setting.

The potted tomato experiment was conducted from October to November 2024 at the Xinxiang Comprehensive Experimental Base of the Chinese Academy of Agricultural Sciences (35°09′N, 113°47′E). The cultivation environment was a greenhouse with potted plants, ensuring no interference from climatic factors. An aluminum alloy frame with dimensions of 120 cm in length, 60 cm in width, and 115 cm in height was set up. Three T8 LED tubes were placed on top of the frame to provide artificial supplementary light (**Figure 1**). The pots used in the experiment had a top diameter of 16 cm, a bottom diameter of 14 cm, and a height of 10.5 cm.

The soil used in the experiment was collected from the 0–40 cm plow layer of the open field. Prior to the experiment, ring knives were inserted at depths of 0–10 cm and 20–30 cm in the soil surface to measure the bulk density and field capacity. The measured field capacity was 21.77% (mass water content), and the soil bulk density was 1.35 g/cm³. The soil was exposed to sunlight for two days and treated with wettable powder fungicide and carbendazim, for sterilization. Afterwards, the soil was naturally air-dried with plant residues and other impurities removed. Then, the soil was packed into pots in batches. The tomato plants (*Solanum lycopersicum L*. cv. ‘Honghongdou’) were transplanted at the four-leaf stage. Management was carried out according to the Organic Food Tomato Facility Production Technical Specifications (Feng, 2021; Lian and Le, 2023) combined with local farmer practices. The fertilization rates for N, P_2_O_5_, and K_2_O were 0.27 g/(kg dry soil), 0.11 g/(kg dry soil), and 0.27 g/(kg dry soil), respectively.

After transplanting the tomato seedlings, normal irrigation was applied on October 6 to ensure their successful adaptation to the environment. After a 16-day period of stable growth, different water stress treatments were initiated on October 22. The stress treatment was continuous and non-cyclical, lasting for 22 days until it ended on November 13. The experimental design was based on the methodology outlined in the literature (Zhuang, 2020) and combined with local management practices. Three soil moisture gradients were established for the experiment, named CK (90%-100% of field capacity), W1 (70%-80% of field capacity), and W2 (50%-60% of field capacity). Soil water content was measured using the oven-drying method, with samples taken daily. When the soil moisture fell below the lower threshold, irrigation was promptly applied to adjust the moisture back to the set value, maintaining the soil moisture within the specified set range. Tomato plants were manually measured once a week, including plant height, number of petioles, and number of leaves.

### 2.2 Data collection and dataset development

**Figure 2:**
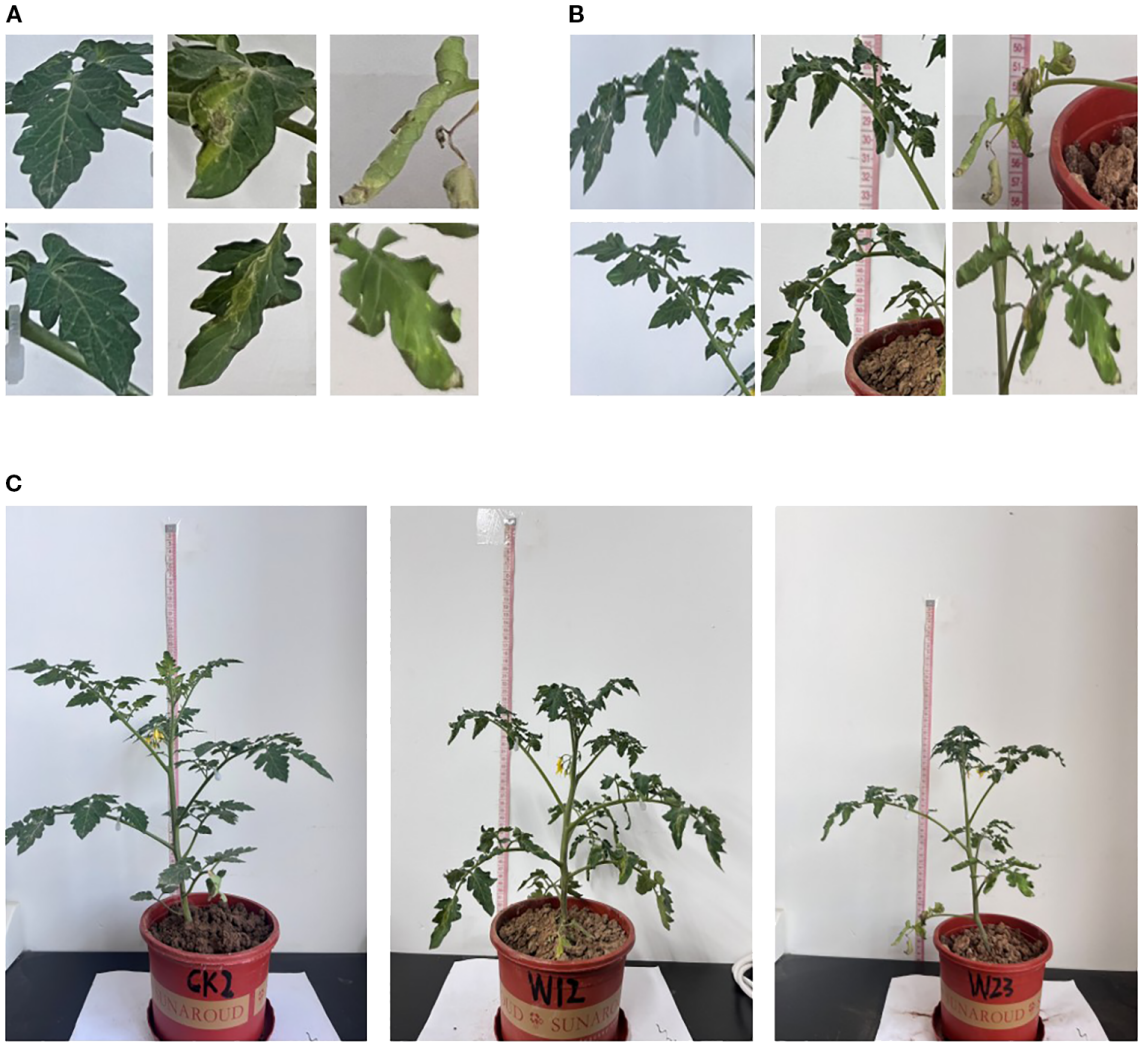
Phenotypic characteristics of tomato under different water stress **(A)** Leaf images; **(B)** Petiole images; **(C)** Overall plant images.

Phenotypic images of the tomato were captured using a smartphone (iPhone 13, 12 MP camera with a dual-camera system, including wide and ultra-wide lenses). The camera was positioned at the same horizontal height as the potted plants, with a horizontal distance of 60 cm during the capture periods. As shown in **Figure 2**, the images were collected to capture the overall characteristics of the tomato during the seedling and flowering-fruit-setting stages, as well as the phenotypic information of the plants. Based on actual conditions, images of the plants were captured twice daily, at 9:00 AM and 6:00 PM, avoiding periods of strong direct sunlight and low light to ensure relatively stable lighting conditions. The image capture used fixed parameters (such as exposure time and white balance) that remained constant throughout the entire collection process. To reduce interference, a white background plate was used for photography.

To develop the dataset, the LabelImg annotation tool (version 1.8.6, YOLO format) was used for manual annotation of the tomato plant phenotypic features. Three different water treatments were applied: CK group (596 images), W1 group (512 images), and W2 group (515 images). The phenotypic features annotated included leaf, height, and petiole. The label format used was “txt,” with label files sharing the same name as the image files. Each file contained five types of information: label_index, x, y, w, and h. The dataset was split into training, validation, and test sets in a 7:2:1 ratio.

### 2.3 Model selection and improvement

**Figure 3:**
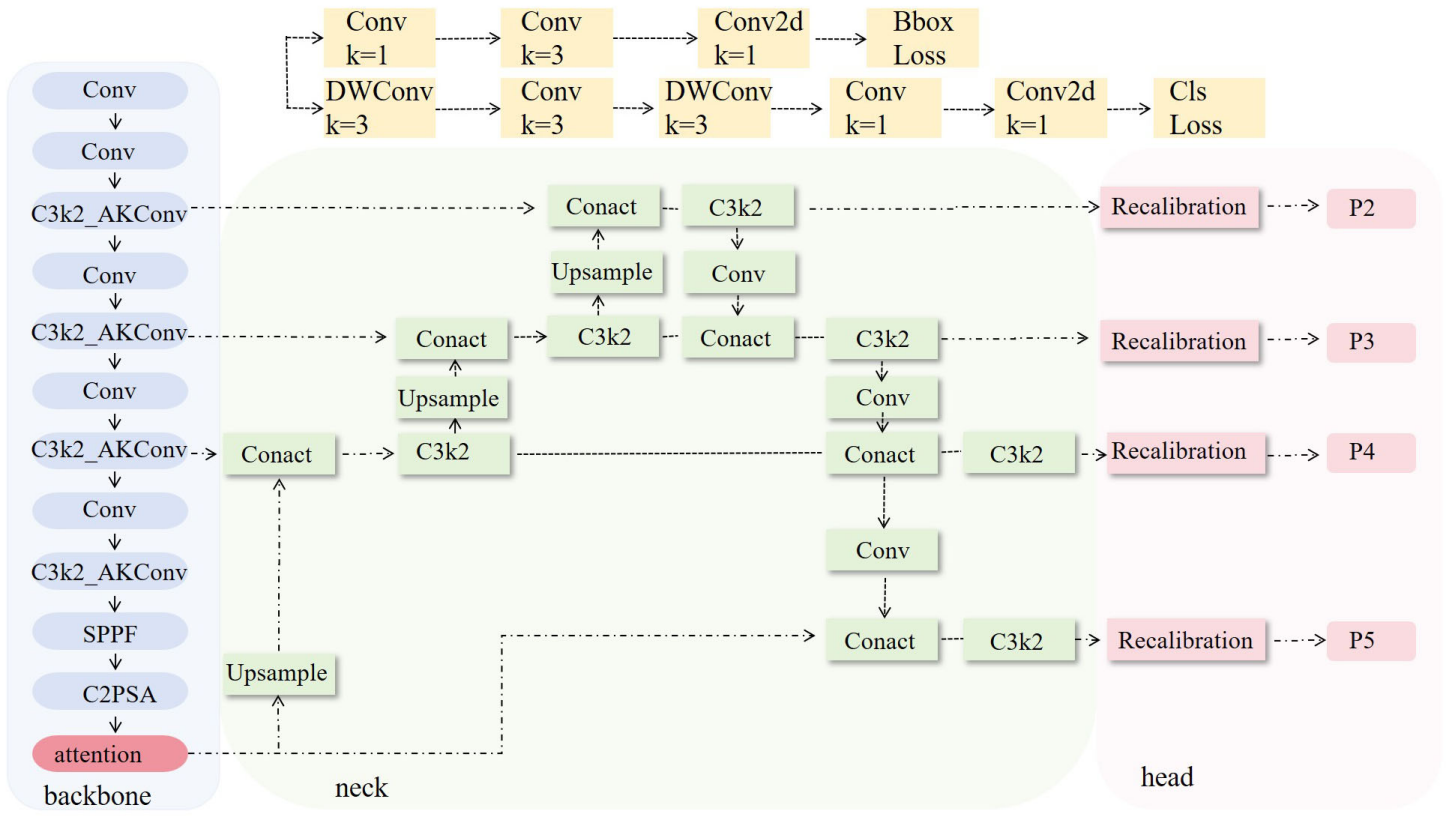
Structure of the improved YOLOv11 model.

To effectively extract phenotypic features of the plants, we introduced a phenotypic detection framework based on the YOLO model. The optimized C3k2 module is integrated into the backbone network to enhance feature representation ability. An attention mechanism is incorporated to improve the model’s ability to capture both local and global information. Additionally, the recalibration FPN-P2345 detection head was used to enhance the accuracy and structural modeling ability for detecting small-scale targets, such as plant height, leaves, and petioles (**Figure 3**). Building on this, a parameter calculation method based on the structural relationships of detection boxes was proposed. By incorporating a calibration board for scale conversion, this method enables the automated, non-destructive extraction of plant height, petiole count, and leaf count. This framework provides stable and reliable technical support for monitoring crop responses due to water stress and conducting quantitative analysis of plant phenotypes.

#### 2.3.1 Model selection

To determine the optimal model, we conducted a performance comparison of five versions of YOLOv11 (YOLOv11n, YOLOv11s, YOLOv11m, YOLOv11l, YOLOv11x). Then, the YOLOv11 series model was selected as the foundational framework for the object detection algorithm in this study. YOLOv11 integrates multi-scale receptive fields, enhanced feature fusion structures, and an improved decoding head, providing excellent recognition capability and high detection accuracy. It is well-suited for tomato phenotypic structure recognition tasks in complex agricultural environments.

The model experiment applied the following environment: Ultralytics version 8.3.9, Python version 3.11.7, PyTorch version 2.1.2, GPU information: NVIDIA GeForce RTX 2080 Ti (11 GB VRAM). To save training time and computational resources, an Early Stopping=30 mechanism was introduced, and in subsequent model training, hyperparameters remained consistent, without loading a pretrained model. The model’s hyperparameters are shown in **Table 1**.

**Table 1:** Model hyperparameters.

Parameter	Value
Epoch	2000
batch size	4
Imgsz	640
lr0	0.01
momentum	0.937
lrf	0.01
Optimizer	SGD

#### 2.3.2 Model improvement

**Figure 4:**
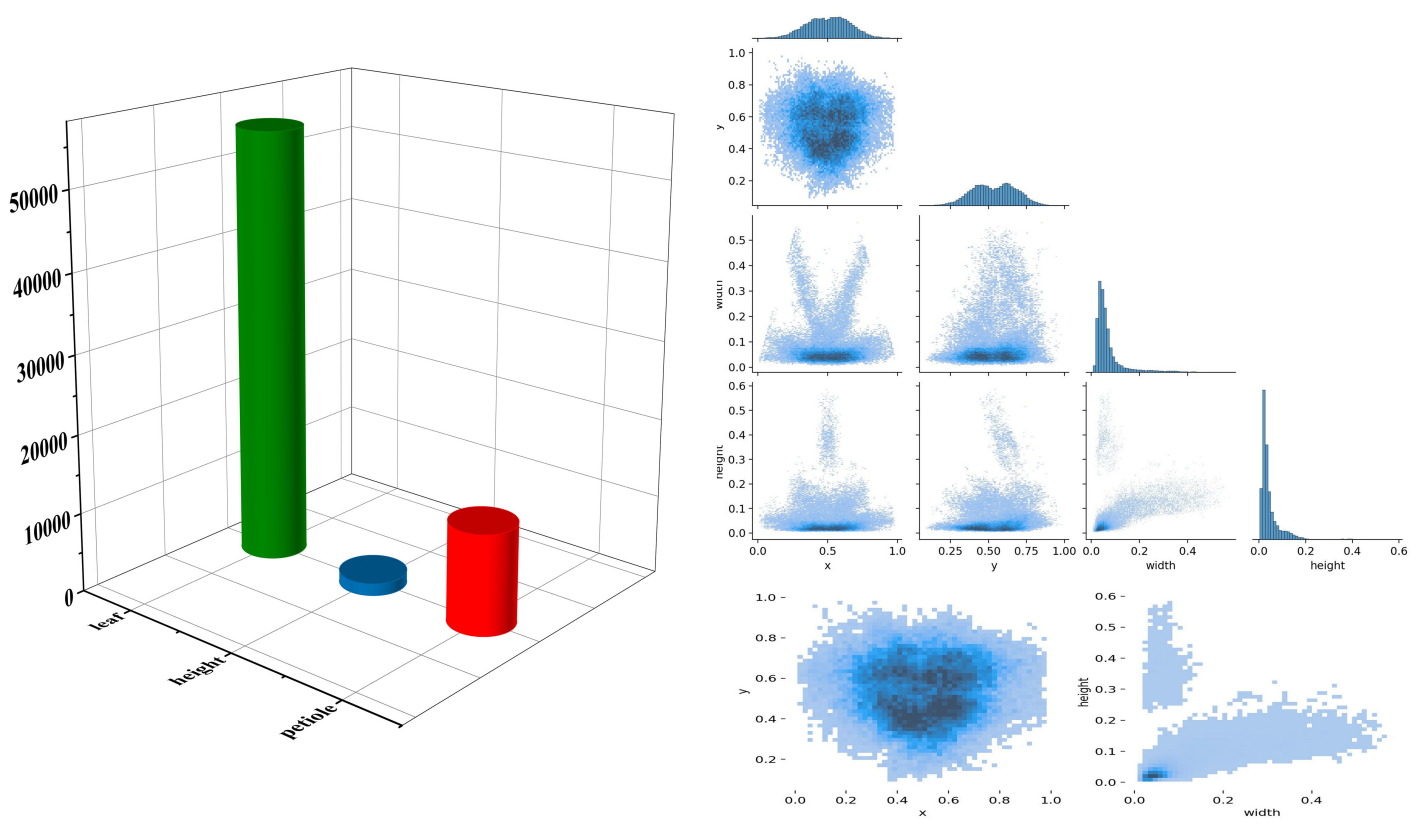
Density map of phenotypic features.

As shown in **Figure 4**, the number of “height” targets is significantly higher than that of “leaf” and “petiole,” indicating a pronounced class imbalance in the dataset, which may adversely affect model training. phenotypic structures such as leaves, petioles, and overall plant height exhibit characteristics of numerous small targets, relatively uniform spatial distribution, and a tendency to be concentrated in the center of the image. Traditional detection models often struggle with incomplete detection and high false positive rates when extracting small and fine-grained structures. Additionally, the target sizes are predominantly small, with a strong positive correlation between width and height, suggesting structural consistency across objects but also highlighting the high proportion of small targets. To improve the model’s ability to perceive multi-scale features of tomato plants, enhance detailed feature modeling, and increase the accuracy of small target detection, attention mechanisms and improved detection head structures were introduced into the backbone of the YOLOv11n model.

##### 2.3.2.1 Improved C3k2 module

**Figure 5:**
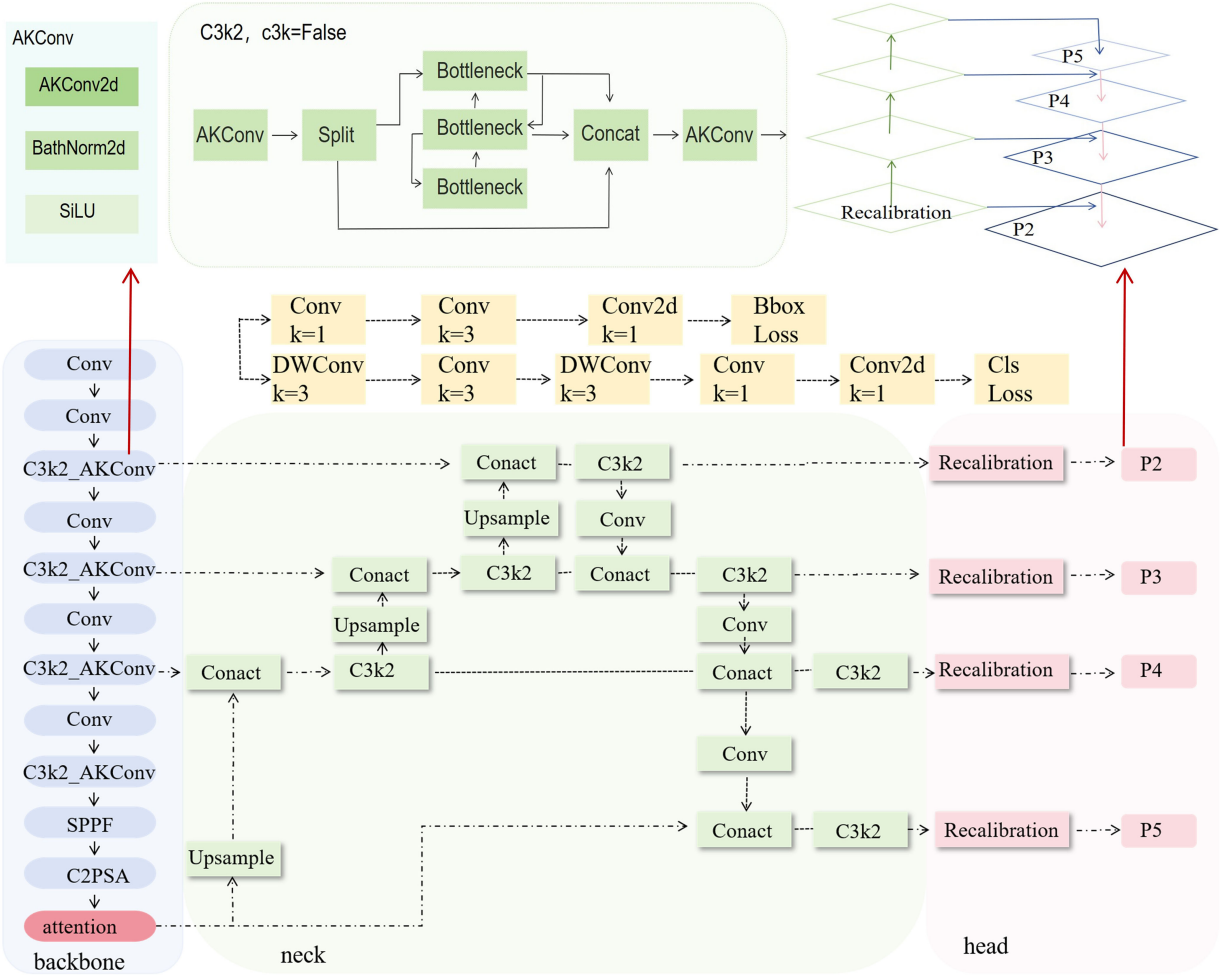
Visualization of improved structure and key modules.


In the backbone network, the C3k2 module is improved to the C3k2_AKConv module (**Figure 5**). This improvement aims to enhance the model’s ability to perceive small-scale and irregularly shaped targets (Zhang et al., 2023). Unlike the fixed structure of standard convolution, the convolution kernel of AKConv (Equation 1) supports an arbitrary number and shape of sampling parameters, allowing it to flexibly adapt to diverse target structures and scale distributions. Overall, AKConv with its variable structure and adaptive characteristics, provides more efficient feature extraction capability, demonstrating superior detection performance, particularly in recognizing small-target phenotypes of tomato.

(1) $$Y=\sum_{i=1}^{\text{n}}\alpha_{\text{i}}\cdot {\text{Conv}}_{\text{ki}}\left(X\right),s.t.\sum_{i=1}^{n}\alpha_{\text{i}}=1$$

where, X represents the input features, Convki denotes the convolution operation using a convolution kernel of size ki, αi is the learnable weight computed by the attention mechanism, and Y is the final output feature after fusion.

##### 2.3.2.2 Add attention mechanism

**Figure 6:**
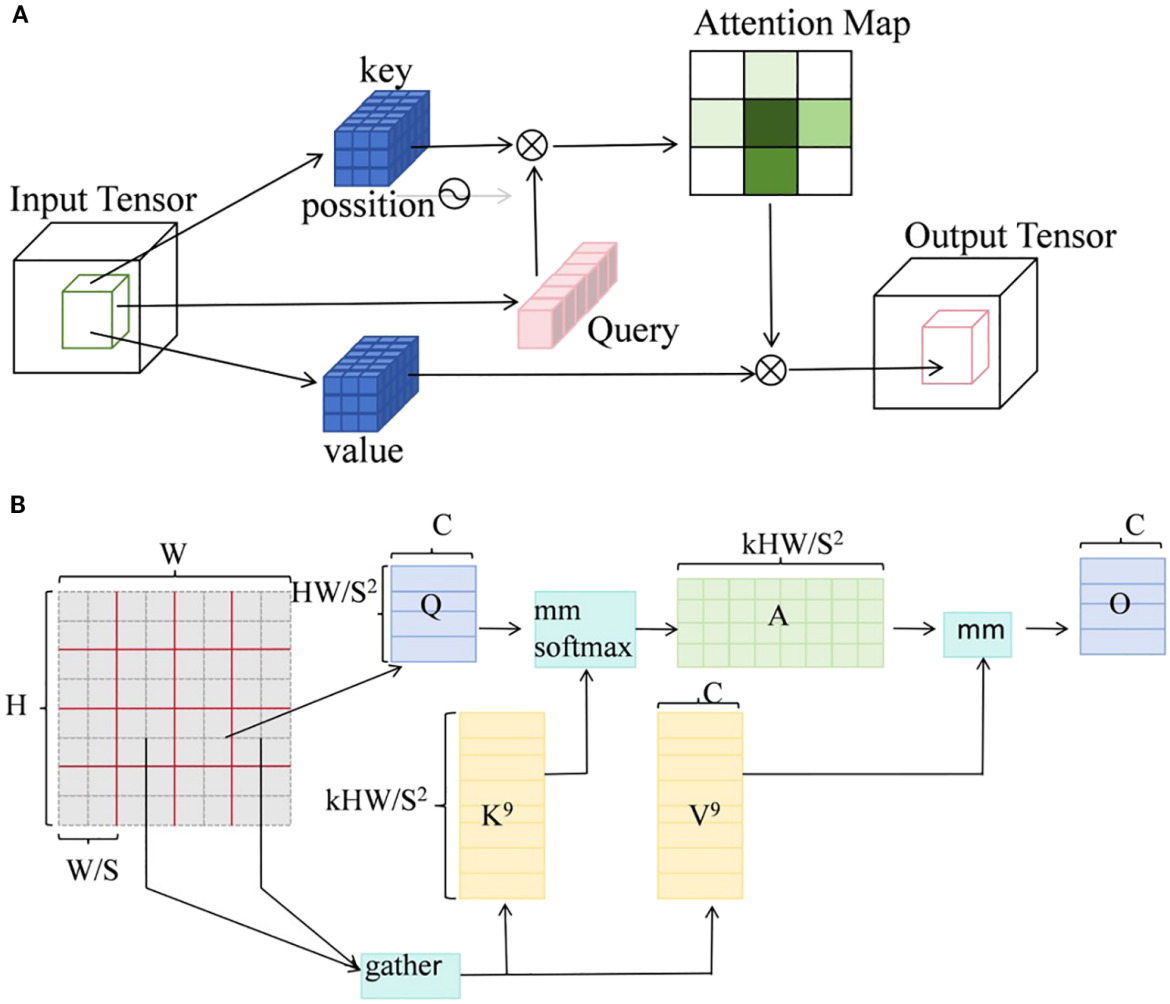
**(A)** Structure diagram of LWA mechanism; **(B)** Structure diagram of BRA mechanism.

To enhance the model’s ability to model small targets and fine-grained structures, two attention mechanisms were introduced in the backbone network: Local Window Attention (LWA, **Figure 6A**) mechanism (Liu et al., 2021)and BiLevel Routing Attention (BRA, **Figure 6B**) mechanism (Zhu et al., 2023).These two attention mechanisms enhance feature extraction capabilities through local perception and cross-region modeling, respectively. They are particularly suitable for detecting complex, small-scale targets, such as leaves and petioles, in crop images.

LWA divides the input feature map into multiple non-overlapping local windows and independently performs self-attention calculations within each window. It significantly reduces the overall computational complexity while enhancing the ability to model local structures (Equation 2). By maintaining computational efficiency, it improves the model’s detection performance for locally dense areas and small-sized targets, making it particularly suitable for extracting fine-grained phenotypic features such as leaves and petioles in tomato plant images.

BRA is widely used in image recognition and object detection tasks. The mechanism is based on a strategy of hierarchical region partitioning and routing information modeling, effectively integrating global modeling capabilities with the efficiency of local representations, It is particularly well-suited for dense object scenarios and small object detection tasks. The BRA attention distribution (Equation 3) significantly improves small target detection accuracy while maintaining model inference efficiency, especially in plant images with blurred edges or densely overlapping areas, demonstrating stronger discriminative ability.


(2) $$Attn\left(Q,K,V\right)=Soft\max\left(\frac{Q_{i}K_{i}^{T}}{\sqrt{d}}\right)V$$

(3) $$Attn\left(Q,K,V\right)=\sum_{i=1}^{N}\pi_{i}\cdot Soft\max\left(\frac{Q_{i}K_{i}^{T}}{\sqrt{d}}\right)V_{i}$$

Where Q, K, and V represent the query, key, and value vectors within the i-th window, respectively, N denotes the number of attention heads or the number of groups, and the weight coefficient of the i-th group. d is the dimension of each head, introduced to prevent excessively large dot product results, which could lead to vanishing gradients in the softmax function, thus serving a normalization purpose.

##### 2.3.2.3 Replace detection head

To enhance the model’s feature representation and localization ability for small and weak-textured targets, such as the edges of tomato leaves and the junctions of petioles, the original detection head structure is replaced with Recalibration FPN-P2345 (**Figure 5**).This structure introduces a feature recalibration mechanism (Lin et al., 2017) based on the traditional Feature Pyramid Network (FPN) (Hu et al., 2020; Dong et al., 2021) and extends the fusion scale to P2–P5 (Lin et al., 2017), enabling full integration from shallow layer details to high-level semantic features, which significantly improves the detection performance of multi-scale targets. Unlike traditional detection heads that only use P3–P5, P2-P5 incorporates the shallow high-resolution feature P2 into the fusion path, allowing the network to maintain global perception capability while enhancing its ability to model edge and texture details for small-sized targets.

The structure mainly consists of the Recalibration module and multi-scale fusion path (P2–P5). The Recalibration module assigns significance weights to feature maps at each layer through a lightweight attention mechanism, enhancing the response of key regions and suppressing redundant background. The multi-scale fusion path uses a bidirectional fusion strategy, combining bottom-up and top-down approaches, to effectively integrate semantic features from different levels, improving feature consistency and semantic expression capability. Overall, this detection head structure effectively improves the model’s detection accuracy in dense regions and fine-grained structures while maintaining computational efficiency. It is particularly suitable for phenotypic feature extraction tasks in crop images, which involve high diversity and significant scale variation.

#### 2.3.3 Performance evaluation metrics

To comprehensively evaluate the performance of each detection model in the task of plant phenotypic feature extraction (Equations 4–8), the following evaluation metrics were adopted in this study: Precision (P), Recall (R), Mean Average Precision (mAP), and Giga Floating-point Operations Per Second (GFLOPs). R measures the proportion of correctly identified positive samples to the total number of actual positive samples. mAP reflects the accuracy and stability of the model’s detection results. GFLOPs are used to evaluate the computational complexity of the neural network.


(4) $$P=\frac{TP}{TP+FP}$$

(5) $$R=\frac{TP}{TP+FN}$$

(6) $$AP=\int_{0}^{1}P\left(r\right)dr$$

(7) $$mAP=\frac{1}{N}\sum_{i=1}^{N}AP_{i}$$

(8) $$FLOPs=2xC_{in}xC_{out}xK^{2}xH_{out}xW_{out}$$

where, TP refers to the number of correct matches between predicted boxes and ground truth boxes; FP is the number of negative samples incorrectly predicted as positive by the model; FN is the number of positive samples incorrectly predicted as negative. P(R) represents the Precision-Recall curve.AP_k_ denotes the Average Precision for the k-th category, and N is the total number of categories. C_n_ and C_out_ represent the number of input and output channels, respectively; K is the kernel size; H_out_ and W_out_ are the height and width of the output feature map. The factor of 2 accounts for both multiplication and addition operations in each convolution.

### 2.4 Phenotypic traits calculation

To quantify the impact of different water stress conditions on tomato plant growth, an automated and non-destructive plant height measurement method was proposed based on the improved object detection model. This method estimates the pixel height by detecting the upper and lower boundaries of the plant stem structure in the image. Also, it uses a calibration board with known dimensions as a scale reference to establish a conversion relationship between pixel values and actual physical measurements, enabling accurate transformation of plant height from image units to real-world units. Specifically, the detection box of the calibration board is extracted from the image at first, and the maximum relative height is selected. Combined with the actual height of the image, the corresponding pixel height Hs is calculated. Based on this, the number of pixels per centimeter can be determined (Equation 9). Subsequently, the detection box of the plant stem is extracted, and its relative height in pixels Hp is calculated. Finally, the actual plant height (PH) is calculated by the following formula (Equation 10).


(9) $$\text{r}=\frac{H_{s}}{1.0}$$

(10) $$PH=\frac{H_{p}}{r}$$

where, r represents the number of pixels corresponding to each centimeter in the image, 1.0 cm is the actual height of the calibration board, PH is the actual plant height, and Hp is the pixel value corresponding to the relative height, in centimeters.

This method relies solely on the detection box position information to perform scale conversion and plant height estimation, without requiring manual intervention or additional equipment. It is suitable for comparing and quantifying the vertical growth status of plants under different water treatment conditions.

Further extraction and analysis of the tomato plant petiole and leaf features under different water stress conditions were conducted. A petiole-leaf association method based on geometric positional relationships was proposed to automatically count the number of leaves on each petiole and calculate the total number of leaves and petioles. First, the categories of leaves and petioles are read from the object detection labels. By extracting the center point coordinates (x, y) and the width and height (w,h) of the detection box, spatial position datasets for both leaves and petioles are constructed. By traversing each petiole bounding box in the detection results, a corresponding 2D rectangular region is constructed. Based on this, it is determined whether a leaf belongs to the corresponding petiole or not. This method can be used to analyze the developmental characteristics of tomato plant organs under different water stress. It helps assess the impact of water stress on leaf distribution and growth structure, providing data support for the correlation modeling between phenotypic parameters and environmental factors.

## 3 Results

### 3.1 The performance of improved model in tomato phenotypic detection

The model training was conducted under a unified dataset and training parameter setup, with the results shown in **Table 2**. YOLOv11x achieved higher recall and mAP50, but its computational complexity reached 195.5 GFLOPs, resulting in high demands on computational resources, slower inference speed (Assunção et al., 2022), more energy consumption, and a relatively large model size. These limitations restrict its performance in practical agricultural environments, especially when deployed on mobile or embedded devices (Ke et al., 2022; Liu et al., 2023). In contrast, the YOLOv11n model, with relatively high accuracy, demonstrated the best overall performance, making it more suitable for deployment on edge devices and for real-time applications in the field.

**Table 2:** Performance comparison of different YOLOv11 models.

Model	Precision/%	Recall/%	mAP50/%	mAP50-95/%	GFLOPs	MB
YOLOv11n	92.5	90.1	92.7	62.5	6.4	5.5
YOLOv11s	93.0	93.0	94.6	66.7	21.6	19.2
YOLOv11m	93.8	93.8	94.9	67.7	68.2	40.5
YOLOv11l	94.4	93.2	94.7	67.0	87.3	51.2
YOLOv11x	93.8	94.0	95.1	68.6	195.5	114.4

To further evaluate the contribution of each improvement module to the model’s performance, an ablation experiment was conducted by sequentially introducing the AKConv convolution, LWA, and BRA attention mechanisms, as well as the detection head module. The experiment focused on key metrics such as detection accuracy and computational complexity, aiming to clarify the impact of each module on small target detection capabilities and overall model efficiency. It provides a basis for optimizing and selecting the final model structure. The results of the ablation experiment are shown in **Table 3**.

**Table 3:** Ablation experiment results.

Model	AKConv	LWA	BRA	P2345	Precision/%	Recall/%	mAP50/%	mAP50-95/%	FLOPS
1	✓	—	—	—	91.7	91.0	93.2	64.7	6.4
2	—	✓	—	—	92.4	90.6	93.3	64.8	6.5
3	—	—	✓	—	92.3	90.9	93.1	64.7	6.7
4	—	—	—	✓	92.8	93.8	95.4	67.3	18.0
5	✓	✓	—	—	92.5	91.3	93.5	64.7	6.4
6	✓	—	✓	—	92.4	89.9	93.0	63.5	6.6
7	—	✓	—	✓	85.2	85.2	86.2	49.4	15.0
8	—	—	✓	✓	84.7	83.2	85.9	48.4	15.1
9	✓	—	—	✓	93.2	94.2	95.4	67.9	17.7
10	✓	✓	—	✓	84.7	85.5	87.1	49.5	14.7
11	✓	—	✓	✓	85.3	83.6	86.0	49.5	15.0

Based on the ablation experiment results (**Table 3**), the performance of each improvement module—AKConv convolution, LWA, BRA attention mechanisms, and RecalibrationFPN-P2345 detection head—was evaluated independently and in combination. Models 1–4 validate the effectiveness of each individual module. Model 4, which incorporates Recalibration FPN-P2345, performed the best and achieved mAP50 and mAP50–95 values of 0.954 and 0.673, respectively. This indicates that the structure significantly enhances multi-scale feature fusion and small target detection. AKConv (Model 1), LWA (Model 2), and BRA (Model 3) all maintained a good balance between precision and computational efficiency, improving mAP50 while keeping FLOPs relatively low.

In the comparison of combined modules, Models 5 and 6 achieved mAP50 values of 0.935 and 0.930, respectively, without increasing computational complexity, demonstrating the synergistic benefits of the attention mechanisms and dynamic convolution in the feature extraction phase. Model 5 highlighted the collaborative advantage of the two techniques in modeling local and adaptive receptive fields, while Model 6 showed slightly lower performance, suggesting some structural overlap between the two, limiting their synergistic effect. On the other hand, combining the P2-P5 detection head with the attention mechanisms in Models 7 and 8 enhanced the depth of semantic fusion but led to a slight decrease in precision. This decrease is likely to be due to information redundancy or conflicts between the attention mechanisms and the high-dimensional feature fusion paths, suggesting that further structural adjustments are needed.

The results of Models 10 and 11 indicated that when all three improved modules were integrated simultaneously, there may be some redundancy or interference during the feature extraction and fusion stages. In particular, the local attention mechanisms (LWA/BRA) combined with deep fusion paths could lead to repeated or conflicting feature information, affecting the overall feature consistency and causing a decrease in detection accuracy. Additionally, the FLOPs of these models were significantly higher than the basic combined schemes, reaching 14.7 and 15.0, respectively. It should be noted that the performance improvement is not substantial while computational resource consumption increases, and may even result in performance degradation.

**Figure 7:**
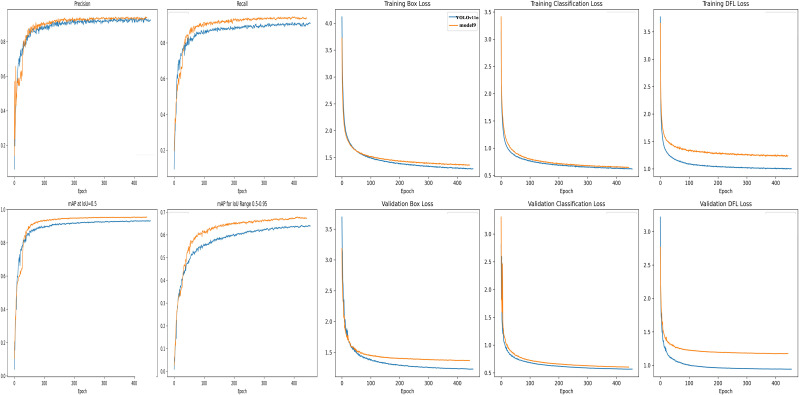
Comparison of the model accuracy.

The model combining AKConv and the detection head module, Model 9, achieved the highest values in both precision and recall (Precision 0.932, Recall 0.942, mAP50 0.954, mAP50-95 0.679), validating the strong coupling between AKConv’s feature extraction advantages and the P2345 detection head’s multi-scale recalibration structure. According to the comparative analysis of the ablation study, each improved module demonstrated independent performance gains. Moreover, Model 9, which combines AKConv and the detection head module, achieved the best overall performance. Comparing the precision, recall, and mAP50 curves of Model 9 and YOLO11n over the entire training process, it fully demonstrated that the improved Model 9 significantly enhanced the extraction of tomato plant phenotype features (**Figure 7**).

**Figure 8:**
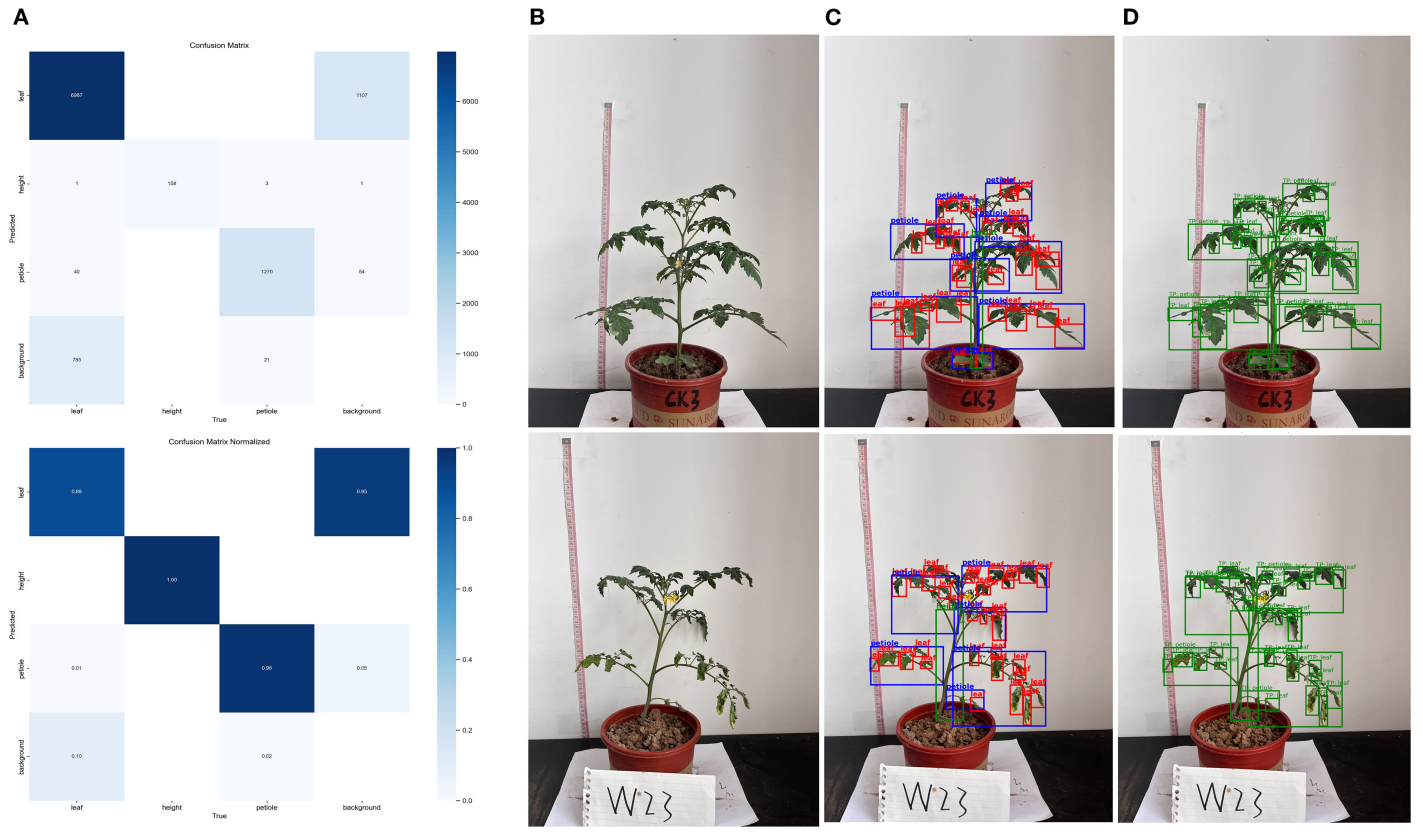
Evaluation of plant trait recognition performance using confusion matrix and object detection **(A)** Confusion matrix and normalized confusion matrix; **(B)** Original tomato plant image; **(C)** Detection box results for plant trait recognition; **(D)** Final prediction results for plant trait recognition.

The results of the normalized confusion matrix (**Figure 8A**) indicate that the model exhibited strong classification performance across most phenotypic categories, with particularly outstanding performance on the height class, suggesting that this trait possesses distinct structural features that make it easier for the model to distinguish. However, confusion was observed between the leaf and petiole classes, likely due to morphological overlap or blurred boundaries. The classification accuracy for the leaf class reached 89%, with 10% misclassified as background, possibly caused by edge ambiguity or background interference. For the petiole class, the accuracy was 98%, with misclassifications mainly to background (5%) and leaf (1%). Two randomly selected samples (**Figures 8B–D**) further illustrate the model’s detection performance under different water treatments. The model effectively captured changes in leaf and petiole distribution, and the recognition results closely matched the phenotypic differences caused by water stress, demonstrating the model’s robustness and biological interpretability.

### 3.2 The performance of improved model in tomato phenotypic traits calculation

#### 3.2.1 Phenotypic traits calculation

To provide a more intuitive illustration of the model performance, 10 representative samples were randomly selected from the total of 86 and are presented in **Table 4**. Additionally, the errors for all samples were visualized to comprehensively reflect the model’s prediction performance across the entire dataset. In this study, the improved YOLOv11 model was primarily used to efficiently recognize and extract key plant information from images. Based on this, geometric analysis of the detection boxes was performed through post-processing, which was then used to calculate the phenotypic parameters of the plants. The results showed that the model measurements were in good agreement with the manually measured values for plant height and petiole count. The average relative error for plant height was 6.9%, and the average relative error for petiole count was 10.12%, both of which fall within an acceptable range. This indicates that the phenotypic analysis framework, combining deep learning object detection and image computation methods, can achieve relatively accurate crop phenotypic parameter extraction without relying on manual intervention, demonstrating high feasibility and practical applicability.

**Table 4:** Comparison of plant height measurement results.

Id	height/cm	petiole/cm	height_pre/cm	petiole_pre/cm	REh/%	REp/%
1	20.5	9.0	24.2	9.0	1.8	0
2	20.0	7.0	20.6	7.0	3.1	0
3	27.5	10.0	28.0	8.0	1.9	20
4	23.0	7.0	23.1	6.0	0.6	14.3
5	27.0	11.0	28.9	11.0	7.3	0
6	35.0	10.0	34.1	10.0	2.7	0
7	30.0	10.0	29.9	8.0	0.6	20.0
8	38.0	10.0	38.4	9.0	1.1	10.0
9	24.0	7.0	23.1	6.0	3.9	14.3
10	28.5	9.0	26.4	8.0	7.6	11.1

**Figure 9:**
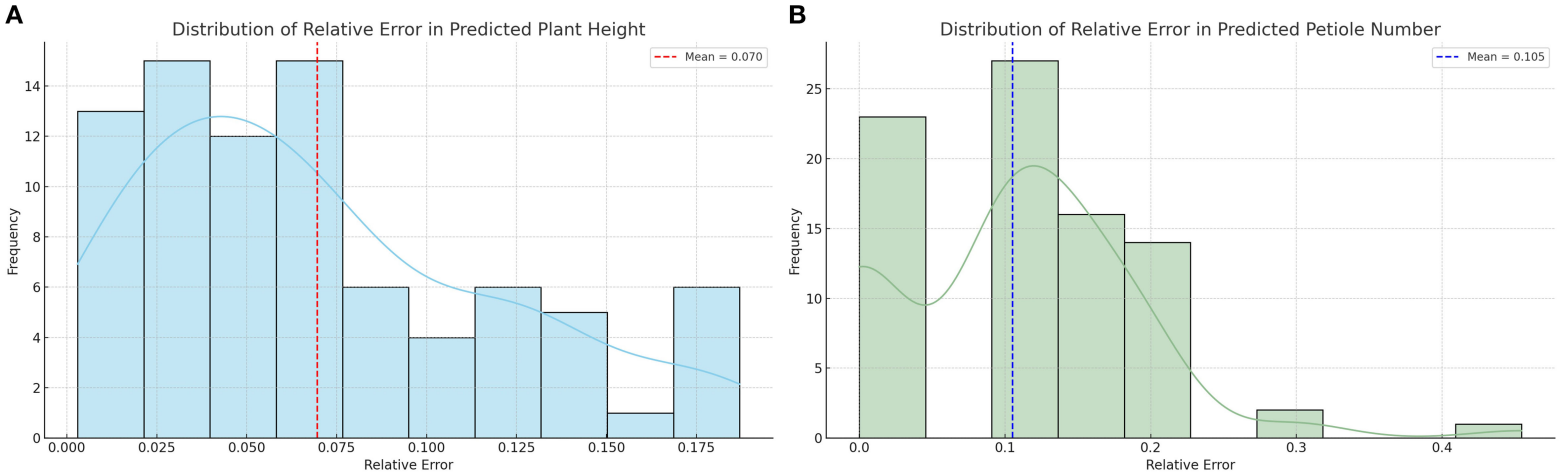
Distribution of relative errors in plant trait predictions **(A)** Distribution of relative error in predicted plant height; **(B)** Distribution of relative error in predicted petiole number.

To comprehensively evaluate the prediction performance of each treatment group for plant height and petiole count, four metrics—coefficient of determination (R²), Pearson correlation coefficient (r), root mean square error (RMSE), and mean absolute error (MAE)—were calculated, and the corresponding visualizations are presented in **Figure 9**. Under different water treatment conditions, the modeling performance of tomato plants exhibited notable differences between the two phenotypic traits: plant height and petiole count. Overall, plant height—as a continuous variable—demonstrated high prediction accuracy under well-watered conditions, with both the coefficient of determination (R²) and the Pearson correlation coefficient (r) reaching relatively high values, indicating strong agreement between predicted and observed values. However, as water stress intensified, the model’s performance deteriorated significantly; in some treatment groups, R² values even turned negative, and both RMSE and MAE increased markedly, reflecting greater variability in plant height under stress conditions. In contrast, petiole count, as a discrete structural trait, exhibited consistently weaker predictive performance across all treatments, characterized by low R² values, high RMSE and MAE, and unstable correlation—some groups even showed negative correlations. This suggests that petiole count is more susceptible to nonlinear influences, making it difficult to model effectively using a unified approach. Therefore, jointly modeling plant height and petiole count may offer a more comprehensive representation of plant responses to water stress and improve the robustness and generalizability of phenotypic predictions.

Due to the large number of tomato plant leaves, as well as their overlapping and complex shapes, manual counting faces significant challenges. It is not only time-consuming and labor-intensive but also prone to omissions or duplicate counts, which can affect the accuracy of the results. Therefore, no manual measurements of leaf count were conducted in this simulation, and only the model’s predicted results were retained as a reference.

#### 3.2.2 Significance Analysis

**Figure 10:**
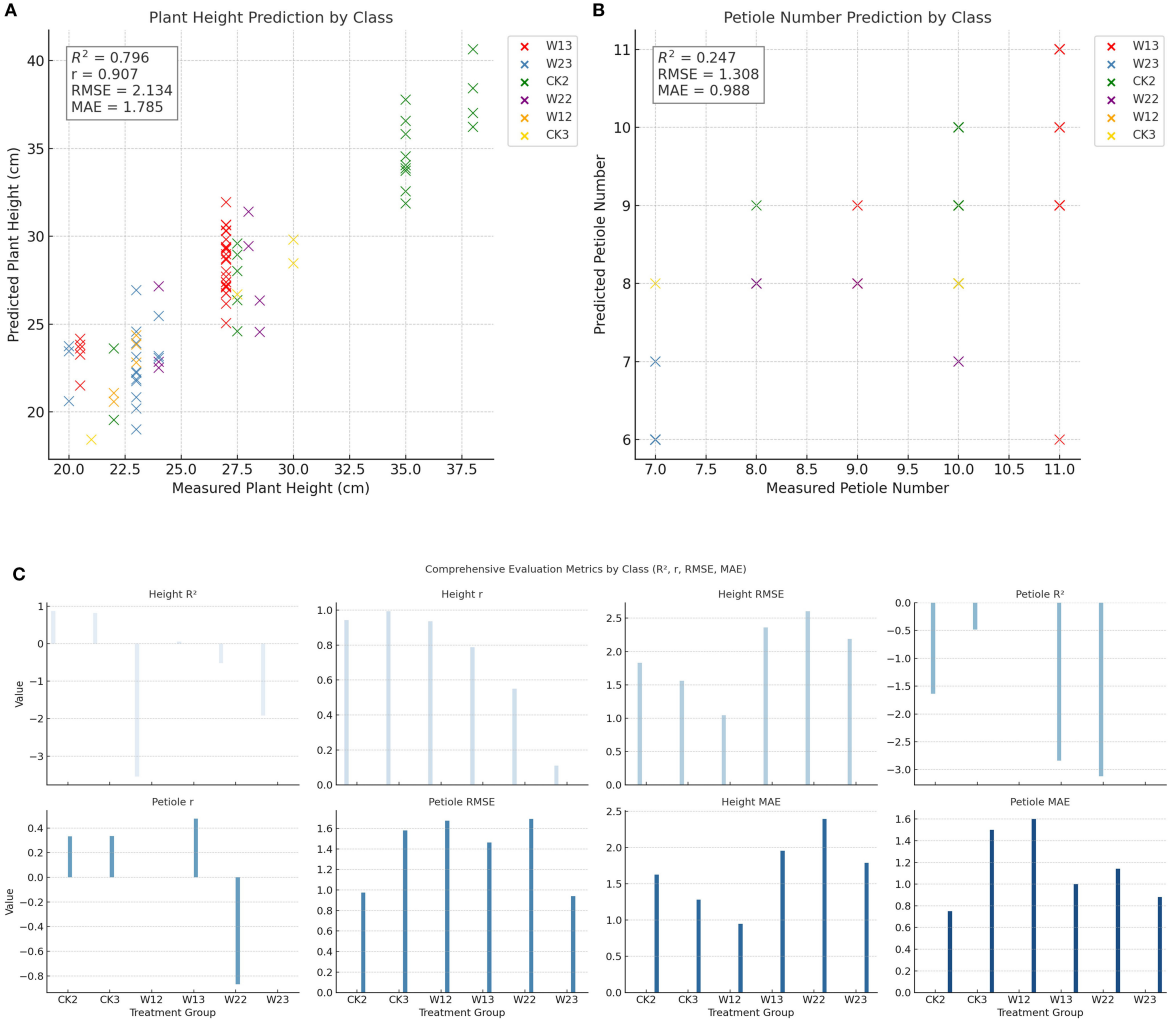
Trait-wise predictive modeling evaluation: R², RMSE, and MAE across Groups **(A)** Plant height prediction by treatment group; **(B)** Petiole number prediction by treatment group; **(C)** Comprehensive evaluation metrics across treatment groups (R², r, RMSE, MAE).

To evaluate the effects of different water stress treatments on the phenotypic traits of tomato plants, this study conducted a systematic statistical analysis using plant height and petiole count as core indicators. Given the assumptions of normality and homogeneity of variances required by traditional one-way ANOVA, preliminary tests were performed to examine the distributional characteristics of the data. The Shapiro–Wilk test indicated that most treatment groups did not significantly deviate from normality for both plant height and petiole count, although slight skewness was observed in certain groups. Further analysis using Levene’s test revealed significant heterogeneity of variances in plant height across treatment groups (*p = 0.012*), suggesting that the assumptions of ANOVA were not fully satisfied with this dataset. In response to these violations, a more robust non-parametric method was employed for group comparisons. The Kruskal–Wallis H test showed highly significant effects of water treatment on both plant height (*p =1.23×10^-8^*) and petiole count (*p = 4.40×10^-^¹²*). To simultaneously evaluate the response patterns of multiple traits, a multivariate analysis of variance (MANOVA) was conducted, revealing statistically significant differences among treatment groups in the combined variables (plant height + petiole count), as indicated by Wilks’ Lambda (*p< 0.0001*). To further identify the sources of variation between groups, pairwise comparisons were performed using the Mann–Whitney U test. The pairwise comparison results were visualized using a heatmap (**Figure 10**), where the color intensity reflects the magnitude of the p-values, providing an intuitive depiction of the distribution of significant differences. Overall, water stress treatments had a significant impact on both plant height and petiole count, with distinct and systematic response differences observed across multiple indicators among treatment groups. The integration of non-parametric tests and multivariate approaches not only enhanced the robustness of the analysis but also improved the reliability of statistical inferences.

**Figure 11:**
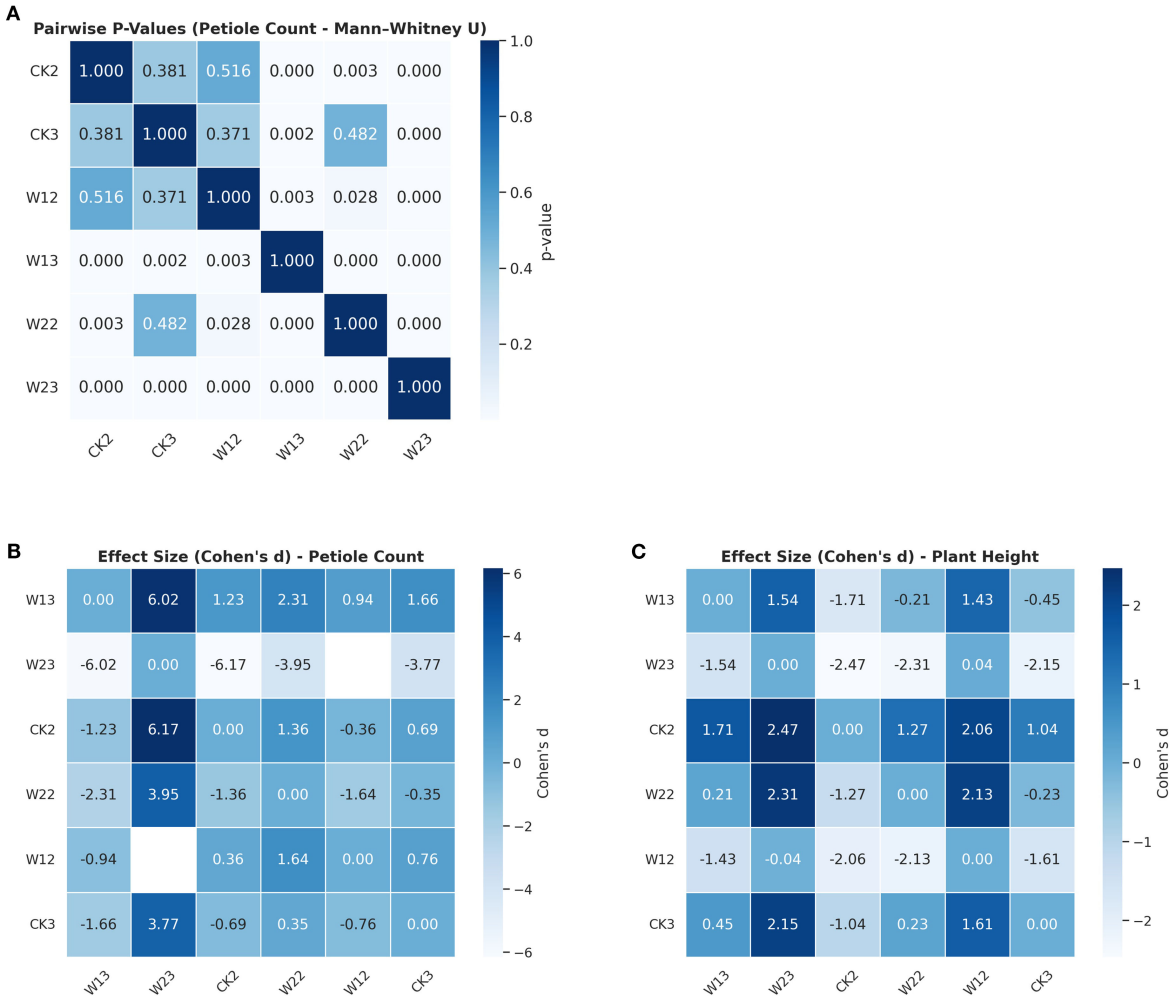
Differences in petiole count and plant height across treatment groups **(A)** Pairwise significance test (Mann–Whitney U) p-values for petiole count; **(B)** Effect size (Cohen’s d) for petiole count; **(C)** Effect size (Cohen’s d) for plant height.

Cohen’s d effect size offers a more intuitive measure of the strength of between-group differences and facilitates the interpretation of trait “sensitivity” rankings under water stress. As shown in **Figure 11**, both plant height and petiole count exhibited high sensitivity to water treatment, with most pairwise comparisons demonstrating strong effects (d>0.8). By quantifying the magnitude of these differences, Cohen’s d provides a more substantive interpretation of treatment effects, reinforcing the conclusion that both traits respond strongly to drought stress. Moreover, it offers a robust quantitative basis for trait sensitivity ranking and supports future investigations into underlying physiological mechanisms.

In summary, p-value analysis provided statistical evidence of significant differences, while Cohen’s d further quantified the practical magnitude of these differences. The combination of both approaches indicated that plant height and petiole count are highly sensitive phenotypic indicators in response to water stress. This integrated analysis not only strengthens the robustness of the statistical conclusions but also offers a quantitative foundation for future investigations into stress response mechanisms and for the screening of drought-resistant cultivars.

### 3.3 Classification of weighted phenotypic traits under different water stress

This study collected phenotypic data of tomato plants subjected to different water stress treatments, with a primary focus on two key traits: plant height and petiole number. To more comprehensively reflect the growth status of the plants, a composite feature was constructed using a weighted linear combination of these two traits, which served as the input for classification modeling. During the model training phase, 80% of the samples were used for training and 20% for testing. The input features were standardized to eliminate the influence of differing feature scales on model performance. To investigate the effect of different weighting schemes on classification accuracy, five combinations of petiole number and plant height weights were defined: 0.5–0.5, 0.6–0.4, 0.7–0.3, 0.8–0.2, and 0.9–0.1.

The input variables primarily consisted of structured phenotypic features, with a limited number of traits and samples. Given that the dataset size does not support the advantages of deep learning models, traditional machine learning algorithms were employed to reduce the risk of overfitting while offering greater model interpretability. Accordingly, seven classical machine learning classifiers were selected: Logistic Regression (LR) and Support Vector Machine (SVM), which are effective in handling linear and high-dimensional data; Random Forest (RF) and Gradient Boosting (GB), representing ensemble learning methods; Decision Tree (DT) and K-Nearest Neighbors (KNN), known for their robustness and interpretability on small to medium-sized structured datasets; and Naive Bayes (NB), which performs well when conditional independence between features is approximately satisfied and provides a useful benchmark for comparison. Each model was iteratively trained, and the classification accuracy on the test set was evaluated and compared (**Table 5**).The key hyperparameter settings for each model are summarized in **Table 6**.

**Table 5:** Comparison of classification models for various phenotypic feature weight combinations.

Models	0.5-0.5	0.6-0.4	0.7-0.3	0.8-0.2	0.9-0.1
LR	0.66	0.55	0.39	0.36	0.27
SVM	0.82	0.65	0.65	0.63	0.52
RF	0.98	0.97	0.98	0.97	0.98
DT	0.98	0.96	0.97	0.96	0.97
KNN	0.98	0.91	0.93	0.90	0.98
NB	0.79	0.73	0.63	0.65	0.27
GB	0.97	0.96	0.98	0.96	0.97

**Table 6:** Main hyperparameter settings of different machine learning models.

Model name	Python class name	Hyperparameter
Logistic Regression	LogisticRegression()	penalty=‘l2’, solver=‘lbfgs’, max_iter=100
Support Vector Machine	SVC(probability=True)	kernel=‘rbf’, C = 1.0, gamma=‘scale’
Random Forest	RandomForestClassifier()	n_estimators=100, max_depth=None, criterion=‘gini’
Decision Tree	DecisionTreeClassifier()	criterion=‘gini’, max_depth=None
K-Nearest Neighbors	KNeighborsClassifier()	n_neighbors=5, metric=‘minkowski’
Naive Bayes	GaussianNB()	Under the assumption of Gaussian distribution
Gradient Boosting	GradientBoostingClassifier()	learning_rate=0.1, n_estimators=100, max_depth=3

**Figure 12:**
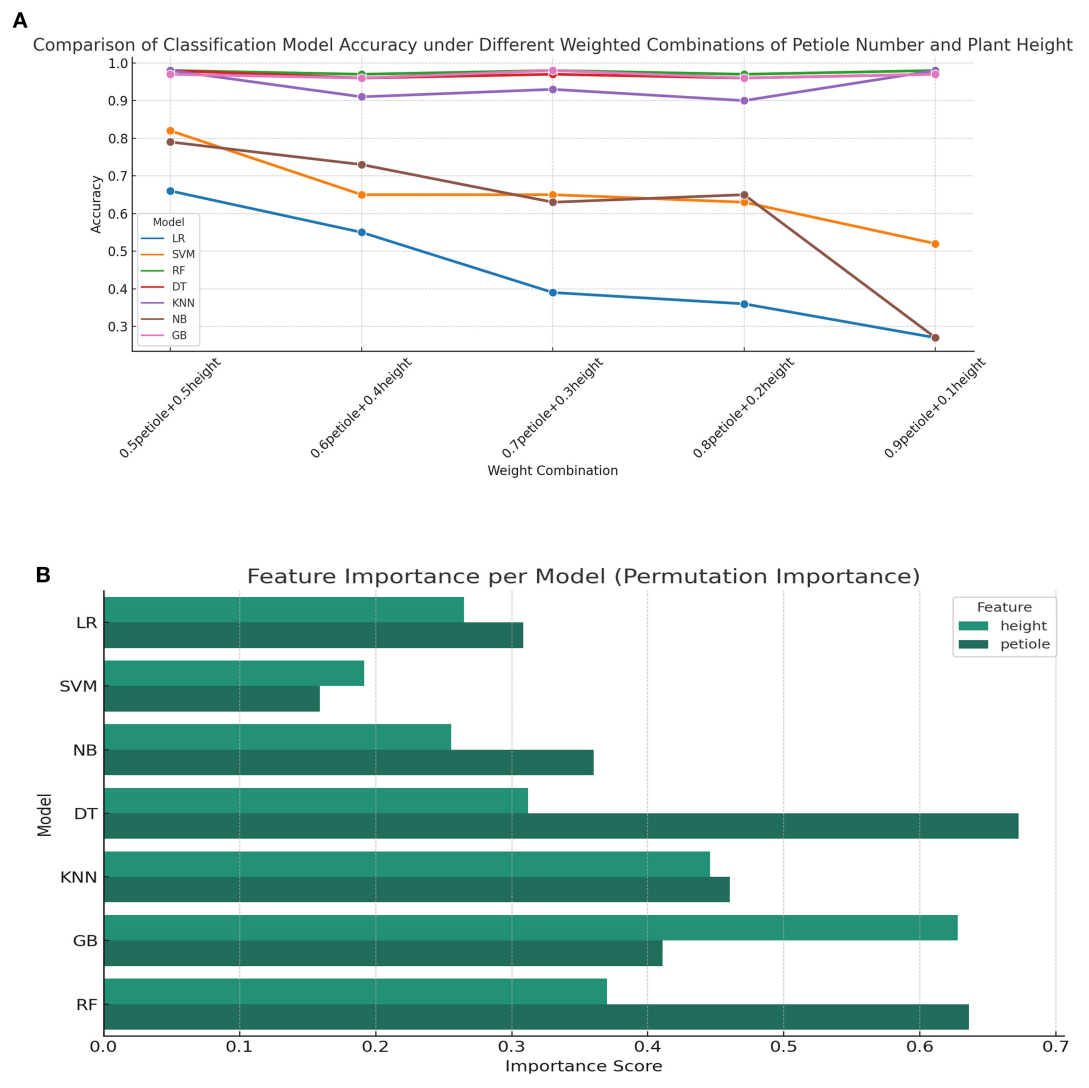
Classification accuracy and feature importance analysis under varying feature weight combinations **(A)** Comparison of classification model accuracy under different weighted combinations of petiole number and plant height; **(B)** Feature importance analysis per classification model.

**Figure 12A** illustrates the classification accuracy trends of seven mainstream models under different weighted combinations of phenotypic features. It can be observed that tree-based models such as Random Forest (RF), Decision Tree (DT), and Gradient Boosting (GB) maintain consistently high accuracy across all combinations, with values approaching saturation (>0.95), demonstrating strong robustness to changes in feature proportions. In contrast, the accuracy of Logistic Regression (LR) and Naive Bayes (NB) drops sharply as the weight assigned to the petiole feature decreases, indicating a stronger reliance on this feature during modeling. The performance of the K-Nearest Neighbors (KNN) model fluctuates moderately with feature weighting but remains relatively high overall, suggesting moderate sensitivity. **Figure 12B** presents the feature importance comparison across models based on the permutation importance method. Overall, tree-based models (DT, RF, GB) exhibit a significantly greater reliance on the height feature than on petiole. In contrast, LR and Support Vector Machine (SVM) show more balanced importance between the two features. NB and KNN models display relatively equal contributions from both features, reflecting a more balanced sensitivity to the two inputs. **Figures 12A, B** complement each other: the former reveals how model performance responds to variations in feature weighting, while the latter provides insight into the underlying feature dependency structure that explains such performance changes.

## 4 Discussion

This study proposes a phenotypic analysis framework for tomato plants that integrates deep learning-based recognition, phenotypic parameter computation, and machine learning-based classification. The framework enables automatic identification, quantitative calculation, and stress-level classification of phenotypic traits under varying water stress conditions. By incorporating the AKConv convolutional module and the Recalibration FPN-P2345 detection head, the model’s perception and localization capabilities for key phenotypic structures—such as plant height, leaves, and petioles—were significantly enhanced. While maintaining low computational complexity, the model also achieved notable improvements in detection accuracy and robustness. Specifically, the optimized model yielded an approximate improvement of 2.7% in mAP@0.5 and 5.4% in mAP@0.5:0.95, demonstrating strong overall performance. Experimental results confirm that the model can achieve automated and non-destructive extraction and computation of tomato phenotypic parameters under different water stress scenarios. Compared with 3D imaging approaches (Tong, 2022; Li, 2023), the proposed method is more lightweight and efficient, offering greater practicality and deploy ability. This study provides a novel approach for evaluating plant water status based on intrinsic phenotypic traits, thereby expanding the application potential of lightweight deep learning models in smart irrigation management.

This study focuses on structural trait changes occurring during the crop growth period and explores their potential for water stress identification. Drought conditions often induce a series of phenotypic adjustments, such as reduced plant height growth rate and altered petiole posture (). The image recognition system proposed in this study is capable of accurately capturing these subtle yet critical changes. This finding expands the applicability of image-based functional phenotyping methods in agricultural practice. The model exhibited consistently high classification performance across various phenotypic weight combinations, further confirming the differential sensitivity of structural traits to water stress and providing a basis for future development of adaptive weighting strategies. Moreover, the study promotes a deeper integration of structural phenotyping and classification modeling, offering a framework for constructing more interpretable diagnostic models by systematically analyzing the contribution of individual traits. The proposed methodology also shows strong potential for extension to other crops and stress types.

Overall, the system developed in this study demonstrates both practical applicability and strong biological interpretability, enabling water stress identification based on intrinsic plant features and making it well-suited for smart irrigation management scenarios. Looking ahead, this work lays the foundation for developing a closed-loop smart agriculture system that integrates environmental sensing, phenotypic analysis, and intelligent decision-making. Future experiments that incorporate real-time phenotypic monitoring may enable a shift from reactive to predictive irrigation strategies, thereby enhancing the proactivity and accuracy of agricultural management.

The model exhibited consistently high classification performance across various phenotypic weight combinations, further confirming the differential sensitivity of structural traits to water stress and providing a basis for future development of adaptive weighting strategies. Moreover, the study promotes a deeper integration of structural phenotyping and classification modeling, offering a framework for constructing more interpretable diagnostic models by systematically analyzing the contribution of individual traits. The proposed methodology also shows strong potential for extension to other crops and stress types. Despite the promising outcomes, certain limitations remain in this study. For instance, key physiological indicators such as relative water content (RWC), leaf water potential, and photosynthetic rate were not collected, resulting in a lack of physiological validation for the model outputs. In addition, no comparative experiments were conducted with traditional image processing methods, making it difficult to systematically evaluate the advantages of the proposed model. The experimental samples were collected from a relatively limited range, and the classification of water stress levels was relatively coarse, indicating that the model’s robustness under complex field conditions requires further improvement. Under challenging scenarios such as extreme lighting or overlapping leaves, certain identification errors still occurred, and the underlying mechanisms of these errors have not yet been systematically analyzed. Moreover, the current dataset was primarily collected from a single experimental site during a specific season, resulting in insufficient data diversity and representativeness. This, to some extent, limits the model’s generalizability across different tomato varieties, growing regions, and lighting conditions. Future work may incorporate multi-source data fusion, cross-regional transfer learning, and edge computing technologies to further enhance the model’s robustness and deployment efficiency across different crops, environments, and application scenarios, ultimately supporting the advancement of smart agriculture and the development of stress-resilient breeding systems.

## 5 Conclusion

The accurate extraction of phenotypic parameters based on image recognition and deep learning approach provides a reliable technology for non-destructive, image-based crop water monitoring in smart irrigation. This study validated the effectiveness of a deep learning approach based on the YOLOv11n model for extracting phenotypic traits of tomato plants under varying water stress conditions. By establishing a precise object detection framework, the method not only enables automatic and accurate identification of key traits such as plant height, leaves, and petioles but also facilitates the subsequent calculation of various phenotypic parameters based on the detection results. In sum, the study achieved the following three key conclusions:

1. By integrating the C3k2_AKConv module into the backbone network, an adaptive convolution kernel fusion mechanism was implemented to improve the detection of small-scale and irregularly shaped targets. Additionally, a Recalibration feature pyramid detection head based on the P2 layer (RecalibrationFPN-P2345) was designed to expand feature fusion scope and incorporate a recalibration mechanism, enhancing multi-scale feature integration and small target localization. The improved model demonstrated excellent performance in tomato phenotypic detection, achieving an mAP50 of 0.954 and an mAP50–95 of 0.679, indicating strong generalization across varying scales and complex backgrounds. Supporting simultaneous multi-object recognition, the model efficiently extracts multiple phenotypic parameters in a single pass, significantly improving data collection efficiency and providing robust support for high-throughput plant phenotyping.
2. Plant height and petiole count, as key structural traits, effectively reflect phenotypic changes induced by water stress. The model performed low prediction errors, with mean relative errors of 6.9% and 10.12%, respectively. Statistical tests and effect size analyses further confirmed their high sensitivity to drought stress, demonstrating not only the feasibility and practicality of using these traits for water stress monitoring, but also providing a solid data foundation for subsequent classification models.
3. The phenotypic feature constructed by the weighted combination of plant height and petiole effectively distinguished different water stress conditions. The Random Forest model achieved the best classification performance with an accuracy of up to 98%. This method provides reliable data support for intelligent identification of crop water stress and smart irrigation.

To conclude, the proposed approach in this study is not only suitable for simultaneous extraction of multiple phenotypic traits but also provides a reliable solution for high-throughput phenotyping of large-scale tomato planting, with promising prospects for widespread application in smart irrigation. Future research can further enhance the model’s generalization ability by incorporating image data from multiple regions and cultivars for training, thereby improving its adaptability to diverse planting environments and plant characteristics.

## Data Availability

The raw data supporting the conclusions of this article will be made available by the authors, without undue reservation.
